# EBV-positive small cell neuroendocrine carcinoma of the nasopharynx with cervical lymph node metastasis: a case report and literature review

**DOI:** 10.3389/fonc.2025.1621859

**Published:** 2025-10-30

**Authors:** Jincai Xue, Wenjuan Ma, Xudong Liu, Yunsheng Wang, Xingyue Wang, Zhihu Li, Youxin Tian, Qinjiang Liu, Fang Dong

**Affiliations:** ^1^ Department of Head and Neck Surgery, Gansu Provincial Cancer Hospital, Lanzhou, China; ^2^ Department of Gastroenterology, Gansu Provincial Cancer Hospital, Lanzhou, China; ^3^ Department of Radiotherapy, Gansu Provincial Cancer Hospital, Lanzhou, China

**Keywords:** Epstein-Barr virus, small cell neuroendocrine carcinoma, nasopharyngeal carcinoma, treatment, case report

## Abstract

**Purpose:**

Investigating the diagnosis and treatment of Epstein-Barr virus-positive small cell neuroendocrine carcinomas of the nasopharynx with cervical lymph node metastasis.

**Methods:**

The clinical data of a patient with Epstein-Barr virus-positive small cell neuroendocrine carcinoma of the nasopharynx with cervical lymph node metastasis were retrospectively analyzed, and the relevant literature was reviewed.

**Case presentation:**

A 65-year-old female patient was admitted with a 1-day history of an incidentally discovered right cervical mass. Thyroid color Doppler ultrasonography revealed enlarged lymph nodes in regions II and Va of the right neck with loss of hilar structure (suspicious for metastasis). Magnetic resonance imaging demonstrated marked thickening of the bilateral nasopharyngeal walls and posterior-superior walls, with multiple enlarged lymph nodes in bilateral cervical level II regions (upper jugular chain), radiologically suggestive of metastatic involvement. Following comprehensive preoperative evaluation, the patient underwent concurrent ultrasound-guided core needle biopsy of cervical lymph nodes and endoscopic nasopharyngeal mass biopsy. The patient was pathologically diagnosed with Epstein-Barr virus-positive small cell neuroendocrine carcinoma of the nasopharynx with cervical lymph node metastasis. The patient received two cycles of etoposide-cisplatin induction chemotherapy and one cycle of targeted therapy (nimotuzumab) combined with radical radiotherapy(intensity-modulated radiation therapy, IMRT) to the nasopharyngeal and cervical lymph node regions. Follow-up magnetic resonance imaging demonstrated significant reduction in both the size of the nasopharyngeal tumor and the initially enlarged lymph nodes compared to previous scans. The patient remains on active anti-tumor therapy with ongoing clinical surveillance pending further longitudinal follow-up assessments.

**Conclusion:**

Epstein-Barr virus-positive nasopharyngeal small cell neuroendocrine carcinoma is an extremely rare head and neck malignancy. Identification of this rare tumor is crucial for disease management and patient prognosis.

## Introduction

Neuroendocrine neoplasms (NENs) are rare tumors originating from peptidergic neurons and neuroendocrine cells (1, 2). Neuroendocrine tumors can occur in almost any organ of the body. Approximately 70% of neuroendocrine tumors arise in the gastroenteropancreatic system, while 25% occur in the respiratory system (3, 4). Neuroendocrine tumors occurring in the head and neck region are relatively rare (5, 6). The fifth edition of the WHO Classification of Head and Neck Tumors categorizes NENs into neuroendocrine tumors (NETs) and neuroendocrine carcinomas (NECs) based on variables such as mitotic count (per mm²), proliferation index (Ki67 labeling index), and necrosis. NECs are further subdivided into small cell NECs and large cell NECs according to their cytomorphological features (7). Small cell neuroendocrine carcinoma of the head and neck accounts for only 0.3% of all head and neck tumors, with approximately 10% of these cases occurring in the nasopharyngeal region (8, 9). The most common cancer of the nasopharynx is non-keratinizing undifferentiated carcinoma, which is highly associated with Epstein-Barr virus (EBV) (10). Currently, nasopharyngeal small cell neuroendocrine carcinoma(SCNEC) is considered unrelated to EBV infection (11). SCNEC originating in the nasopharynx is rare, and cases associated with EBV positivity are even more uncommon. To date, such occurrences remain primarily documented in case reports. This case report presents a EBV-positive small cell NEC of the nasopharynx with cervical lymph node metastasis, accompanied by a review of relevant literature.

## Case report

**Figure 1:**
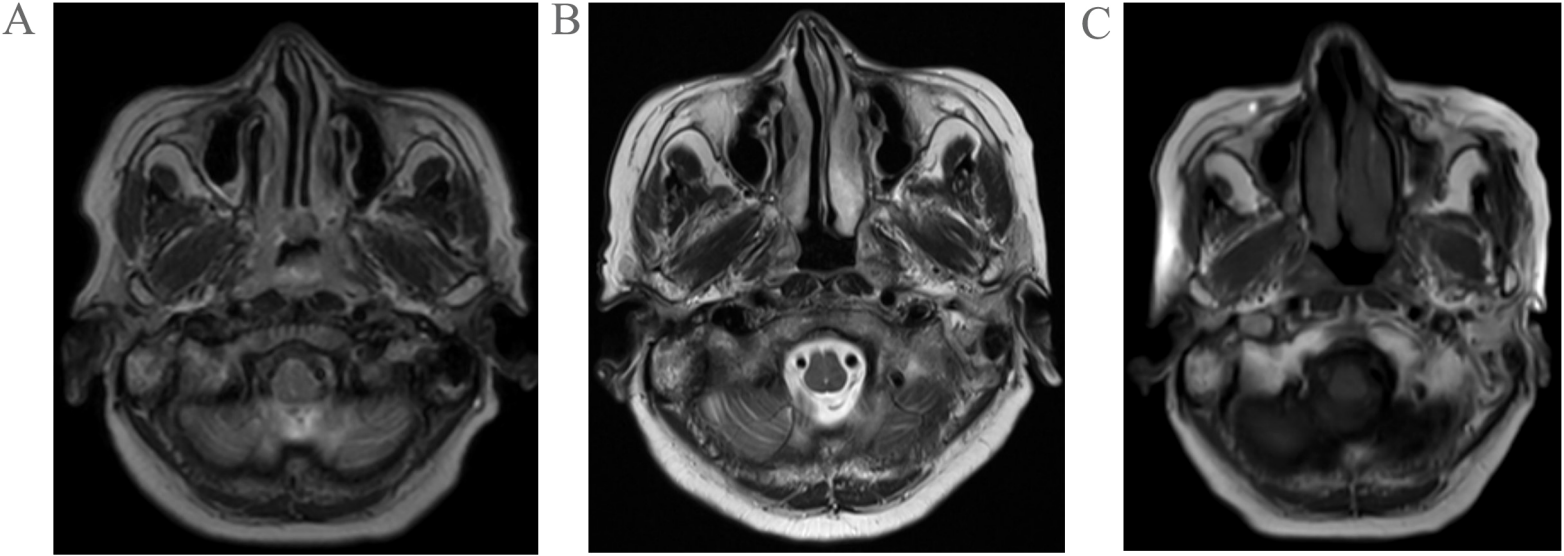
Pretreatment MRI findings of the patient **(A)**; Post-2-cycle chemotherapy MRI findings of the patient **(B)**; Post-1-cycle radiotherapy combined with nimotuzumab targeted therapy MRI findings of the patient **(C)**.

A 68-year-old woman was admitted to the hospital following the incidental discovery of a right cervical mass with a 1-day history. The patient denies any past medical history of diseases, surgeries, or allergies. Physical examination revealed multiple enlarged lymph nodes in the right cervical region. The largest lesion, located in level II, measured approximately 4.0cm×2.0cm, demonstrating firm consistency, non-tenderness, ill-defined borders, and partial mobility. No significant masses were palpated in other regions. Color Doppler ultrasound of the thyroid gland revealed multiple solid nodules in both lobes. The dominant nodule in the left lobe measured 0.3 cm×0.3 cm×0.2 cm, while the largest in the right lobe measured 0.4 cm×0.3 cm×0.6 cm. All nodules were classified as TI-RADS category III. Enlarged lymph nodes were observed in the right cervical level II and Va regions, demonstrating loss of hilar structure (suggestive of metastatic involvement). Quantitative EBV DNA testing revealed an elevated viral load of 1.28×10³ copies/mL (reference range: <4.00×10²copies/mL), suggestive of active viral replication. Magnetic resonance imaging (MRI) demonstrated marked thickening of the bilateral nasopharyngeal walls and posterior-superior walls, with multiple enlarged lymph nodes in bilateral cervical level II regions (upper jugular chain), radiologically suggestive of metastatic involvement(**Figure 1A**). Following comprehensive preoperative evaluation, the patient underwent concurrent ultrasound-guided core needle biopsy of cervical lymph nodes and endoscopic nasopharyngeal mass biopsy.

**Figure 2:**
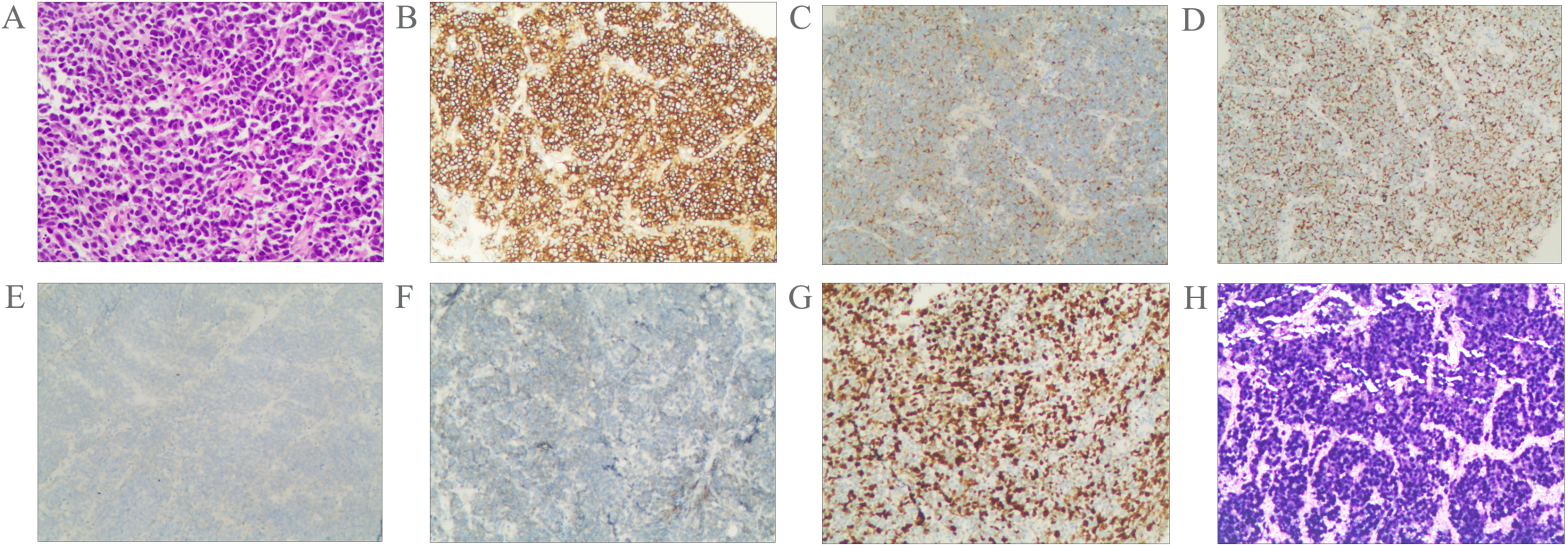
Morphological features of the tumor cells **(A)**; Immunostaining for CD56 **(B)**, Syn **(C)**, CKpan **(D)**, CgA **(E)**, INSM1 **(F)**, Ki-67 **(G)**. The tumor was positive for EBER according to the result of *in situ* hybridization **(H)**. Magnification A-H × 20.

**Figure 3:**
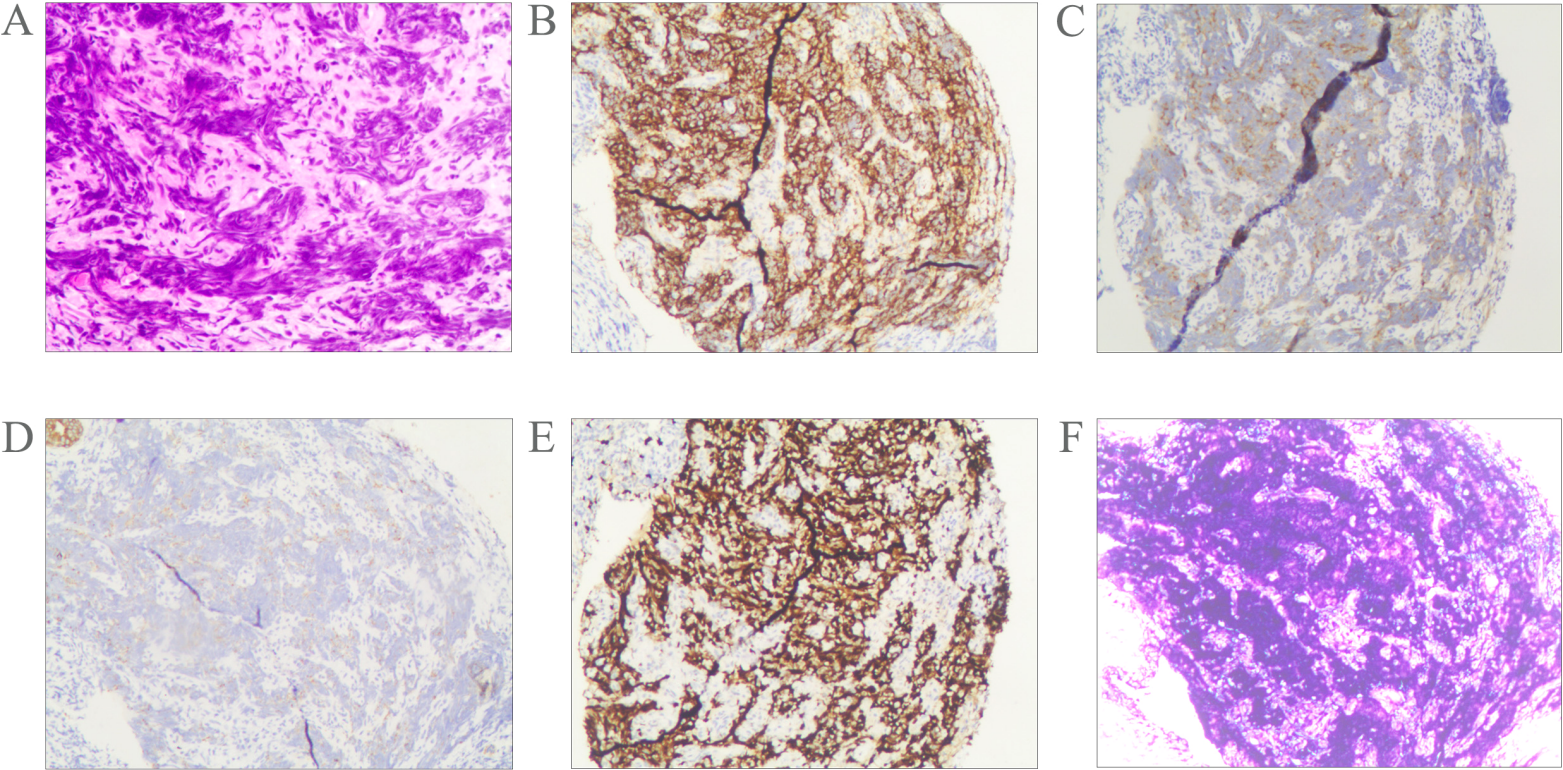
Morphological features of the tumor cells **(A)**; Immunostaining for CD56 **(B)**, Syn **(C)**, CKpan **(D)**, Ki-67 **(E)**. The tumor was positive for EBER according to the result of *in situ* hybridization **(F)**. Magnification A-F × 10.

Pathological examination of fine-needle aspiration biopsy from the right cervical lymph node revealed: Tumor cells are small to medium-sized, arranged in densely nested or sheet-like patterns. The nuclei demonstrate hyperchromasia with granular chromatin and inconspicuous nucleoli. Frequent mitotic figures are observed. The cells exhibit scant cytoplasm and a high nuclear-to-cytoplasmic ratio, with nuclear molding commonly seen. Immunohistochemical staining shows positivity for CD56, Synaptophysin (Syn), and pan-Cytokeratin (CKpan). Weak positivity is observed for Chromogranin A (CgA) and INSM1. Negative staining is noted for S-100 protein, p40, CD3, CD20, NUT, and Thyroid Transcription Factor-1 (TTF-1). The Ki-67 proliferation index is approximately 80%. Epstein-Barr virus-encoded RNAs (EBERs) *in situ* hybridization shows positive results(**Figure 2**). Biopsy of the nasopharyngeal mass reveals: Tumor cells are small to medium-sized, arranged in densely nested or sheet-like patterns. The nuclei are hyperchromatic with granular chromatin and inconspicuous nucleoli. Frequent mitotic figures are identified. The cells display scant cytoplasm and a high nuclear-to-cytoplasmic ratio, with frequent nuclear molding. Immunohistochemical studies demonstrate positivity for CD56 and Syn, with weak positivity for CKpan. Negative staining is observed for p40 and CgA. The Ki-67 proliferation index reaches approximately 90%. EBER *in situ* hybridization is positive (**Figure 3**). Based on the integrated clinical findings, the patient was diagnosed with EBV-associated small cell NEC of the nasopharynx with cervical lymph node metastasis (c-T3N2M0, Stage IVA).

The patient received two cycles of etoposide-cisplatin induction chemotherapy. Follow-up MRI performed at one month post-chemotherapy demonstrated significant regression in both the nasopharyngeal tumor dimensions and initially enlarged lymph nodes compared to pretreatment baseline measurements(**Figure 1B**). Subsequently, the patient underwent one cycle of targeted therapy with nimotuzumab combined with definitive radiotherapy(IMRT) to the nasopharynx and cervical lymph nodes (prescribed doses: primary nasopharyngeal lesion: total dose of 69.96 Gy in 33 fractions; cervical lymph nodes: total dose of 66 Gy in 33 fractions; subclinical disease region of nasopharynx: total dose of 59.4 Gy in 33 fractions;bilateral cervical lymphatic drainage areas: total dose of 54.12 Gy in 33 fractions). Follow-up MRI at 1 month post-radiotherapy demonstrated minimal regression of the primary nasopharyngeal tumor and persistent lymphadenopathy in baseline-involved nodal regions (**Figure 1C**). The patient remains on active anti-tumor therapy with ongoing clinical surveillance pending further longitudinal follow-up assessments.

A comprehensive literature review identified 10 documented cases of EBV-positive nasopharyngeal small cell NEC with cervical lymph node metastases (12–14). The primary clinical characteristics are outlined in **Table 1**.

**Table 1:** Characteristics of reviewed reports of EBV-positive small cell neuroendocrine carcinoma of the nasopharynx with cervical lymph node metastasis.

Author	Year	Country	Number of cases	Age	Sex	Tumor Site	Metastatic site	Stage	EBER	Treatment	Follow-up time/prognosis
Fan CZ (12)	2024	China	1	24	Male	Nasopharynx(posterior and left walls)	Cervical lymph nodes	T2N2M0	Positive	Immunochemotherapy(Cisplatin-Etoposide-Carilluzumab)and radiotherapy	2 months/AWD,PR
Chen Y (13)	2024	China	3	33	Male	Nasopharynx(posterior and bilateral walls)	Cervical lymph nodes	T2N3M0	Positive	Chemotherapy(docetaxel-cisplatin)and chemoradiotherapy(cisplatin-radiotherapy)	15 months/AWD,PR
				39	Male	Nasopharynx(posterior and left walls)	Cervical lymph nodes;liver	T4N1M1	Positive	NA	13 months/AWD,PR
				73	Female	Nasopharynx(posterior and bilateral walls)	Bilateral supraclavicular region;cervical lymph nodes	T3N3M0	Positive	Immunochemotherapy(Carboplatin-Etoposid-Slulizumab)and immunochemotherapy (Carboplatin -Slulizumab) and radiotherapy	11 months/AWD,PR
Zhang XY (14)	2024	China	6	47	Male	Nasopharynx	Lymph node;liver;bone	NA	Positive	Chemotherapy and radiotherapy and cetuximab	5 months/DOD
				27	Male	Nasopharynx	Lymph node	NA	Positive	Chemotherapy and radiotherapy	106 months/NED
				42	Female	Nasopharynx	Lymph node	NA	Positive	Chemotherapy and radiotherapy	18 months/ovaries metastasis; 96 months/NED
				54	Male	Nasopharynx	Lymph node	NA	Positive	Chemotherapy and radiotherapy	NA
				57	Male	Nasopharynx	Lymph node	NA	Positive	Chemotherapy and radiotherapy and immunotherapy	8 months,lung metastasis/AWD
				64	Male	Nasopharynx	Lymph node	NA	Positive	Chemotherapy and radiotherapy	44 months/NED

AWD, alive with disease; DOD: died of disease; NED, no evidence of disease; NA, not available; PR, partial remission.

## Discussion

Nasopharyngeal carcinoma is a rare malignant tumor located in the nasopharynx, characterized by a distinct geographical distribution and is particularly common in Southeast Asia and southern China (15–17). Furthermore, genetic factors, EBV infection, long-term exposure to chemical substances, and lifestyle habits (such as smoking, alcohol consumption, etc.) may all increase the risk of developing nasopharyngeal carcinoma (18). According to the 2022 global cancer statistics, there were a total of 120,416 new cases diagnosed and 73,476 deaths attributed to nasopharyngeal carcinoma (19). In low-risk populations, the incidence of nasopharyngeal carcinoma (NPC) reaches its first peak between ages 18-25, then rises to a second, higher peak around 65–79 years old. In contrast, among high-risk populations, NPC incidence shows a single peak between approximately 45–59 years of age, followed by a plateau phase or moderate decline (20). Nasopharyngeal carcinoma is histologically classified into keratinizing squamous cell carcinoma, non-keratinizing squamous cell carcinoma (including differentiated and undifferentiated subtypes), and basaloid squamous cell carcinoma (21). However, non-keratinizing differentiated type and undifferentiated type nasopharyngeal carcinoma (NPC) are closely associated with EBV (22).

EBV, also known as human herpesvirus 4 (HHV-4), is the first discovered human tumor virus (23). Over 95% of the world’s population is infected with EBV. A significant characteristic of EBV is its ability to establish lifelong latency in humans, which is facilitated by its latent phase (24). The EBERs are expressed in EBV-infected tumor cells. EBER *in situ* hybridization is recognized as the gold standard for detecting latent EBV infection in tissue samples (25, 26). The International Agency for Research on Cancer (IARC) has classified EBV infection as being associated with various malignancies, such as nasopharyngeal carcinoma, Burkitt’s lymphoma, immunosuppression-related non-Hodgkin lymphoma, and others (27). The etiology of head and neck neuroendocrine carcinoma remains largely unclear and is primarily associated with human papillomavirus (HPV) infection. Compared to neuroendocrine carcinomas in other head and neck regions, literature indicates that large cell neuroendocrine carcinoma of the nasopharynx shows a strong correlation with EBV infection (11, 28, 29). Currently, small cell neuroendocrine carcinoma of the nasopharynx is considered to be unrelated to EBV infection (11).

Neuroendocrine carcinoma of the head and neck is a rare malignant tumor, accounting for only 0.3% of all head and neck cancers (30). By definition, it exhibits strong staining for neuroendocrine tumor markers such as synaptophysin, chromogranin A, CD56, CD57, neuron-specific enolase (NSE), and PGP9.5 (31). According to histological classification, head and neck neuroendocrine carcinomas are categorized into small cell neuroendocrine carcinoma, large cell neuroendocrine carcinoma, and mixed neuroendocrine-non neuroendocrine neoplasms (32). In this case report, based on histological changes, immunohistochemical findings, and *in situ* hybridization results, the patient was diagnosed with EBV-positive small cell neuroendocrine carcinoma of the nasopharynx accompanied by cervical lymph node metastasis.

Small cell neuroendocrine carcinoma arising in the nasopharynx is rare, and cases associated with EBV positivity are even rarer. A study revealed that POU2F3 expression was significantly elevated in EBV-positive nasopharyngeal small cell NEC relative to nasopharyngeal small cell NEC. Gene expression profiles also showed increased POU2F3 levels in these EBV-positive cases, indicating a significant association (33). However, in the diagnostic process, it is essential to differentiate it from other malignant tumors originating in the nasopharynx (1):Undifferentiated non-keratinizing squamous cell carcinoma: Tumor cells exhibit large syncytial-like morphology with indistinct cell borders. Nuclei are round or oval with vesicular chromatin and centrally located nucleoli. Immunohistochemistry shows strong positivity for pCK, HCK, p63, and p40. EBV can be detected by EBER *in situ* hybridization in 75-100% of cases (21) (2). NUT carcinoma: Tumor cells are round, oval, or spindle-shaped, exhibiting extensive infiltration and a high mitotic rate. The nuclei appear round or oval. Immunohistochemical staining demonstrates positivity for p63, p40, and CD56. Approximately 30% of cases may show focal squamous differentiation with abrupt keratinization (34, 35) (3). Mucosal melanoma: Tumor cells exhibit spindle-shaped, epithelioid, or plasmacytoid morphology. The cytoplasm is amphophilic or eosinophilic, containing varying amounts of pigment, with prominent nucleoli. In rare cases, the tumor may present with small cell or nevus-like morphology. Immunohistochemical staining shows positivity for SOX10, S-100 protein, HMB45, and Melan A (35, 36) (4). Olfactory neuroblastoma: Tumor cells are small and uniform with a high nuclear-to-cytoplasmic ratio, round or oval nuclei displaying “salt-and-pepper” chromatin, and small or absent nucleoli. Necrosis is absent, and mitotic figures are rare or absent. Homer-Wright pseudorosettes are observed in approximately 30% of tumors, while Flexner-Wintersteiner true rosettes are seen in 5% of cases. Immunohistochemical staining demonstrates positivity for synaptophysin, CgA, CD56, and S-100 protein (37).

Small cell neuroendocrine carcinoma is highly aggressive and has a predilection for extensive local infiltration, including lymphovascular invasion and perineural invasion (38). Furthermore, it is also prone to regional lymph node metastasis and diffuse systemic spread (39). It is reported that the prognosis of small cell neuroendocrine carcinoma of the nasopharynx is the worst among all small cell neuroendocrine carcinomas in the head and neck region (40). Due to the extremely low incidence, rarity, and sporadic nature of nasopharyngeal small cell neuroendocrine carcinoma, there is limited established experience in its treatment. Currently, it is believed that radiotherapy can achieve good local control for small cell neuroendocrine carcinoma of the nasopharynx, while chemotherapy can effectively control distant metastasis of the disease (9). Therefore, it is currently considered that comprehensive treatment combining radiotherapy and chemotherapy should be the first-line therapy for small cell neuroendocrine carcinoma of the head and neck. In recent years, the expanding clinical utilization of immune checkpoint inhibitors and molecular targeting technologies has positioned immunotherapy and targeted therapy as promising therapeutic alternatives in head and neck oncology (41, 42). Nimotuzumab is an EGFR monoclonal antibody that blocks the epidermal growth factor receptor signaling pathway, inhibits tumor angiogenesis, and enhances radiosensitivity, thereby achieving a synergistic effect where the combined outcome surpasses the individual contributions (43). A retrospective study found that the combination of anti-EGFR targeted therapy with IMRT was not inferior to concurrent chemoradiotherapy in terms of survival outcomes, while demonstrating relatively fewer adverse reactions (44). A retrospective analysis of 32 elderly patients with locally advanced nasopharyngeal carcinoma reported that the combination of nimotuzumab and intensity-modulated radiotherapy demonstrated encouraging efficacy and was well-tolerated (45). In this case report, the patient underwent two cycles of etoposide-cisplatin (EP) induction chemotherapy followed by radiotherapy with concurrent molecular targeted therapy (MTT). She is tolerated the therapeutic regimen well and was generally in good condition. Follow-up MRI showed significant regression in both the nasopharyngeal tumor dimensions and initially enlarged lymph nodes compared to pretreatment baseline measurements.

We conducted a systematic review of the 10 previously reported similar cases. All patients underwent imaging studies and pathological examinations for the diagnosis of EBV-positive nasopharyngeal small cell neuroendocrine carcinoma. Five patients received chemotherapy combined with radiotherapy, one patient received trimodal therapy incorporating chemotherapy, radiotherapy, and targeted agents, while three cases underwent immunochemotherapy and radiotherapy. All patients’ tumor foci demonstrated partial response (PR) following treatment. In our case, the patient received chemotherapy and targeted therapy combined with radiotherapy, with tumor foci demonstrating PR observed during follow-up. At the time of writing this report, the follow-up period was 5 months.

## Conclusion

EBV-positive nasopharyngeal small cell NEC is an extremely rare head and neck malignancy characterized by atypical clinical manifestations, high-grade aggressiveness, and elevated rates of both locoregional and distant metastasis. Therefore, the diagnostic workup requires comprehensive integration of clinical data, histopathological features, immunophenotypic profiles, and EBER *in situ* hybridization testing to minimize the risk of misdiagnosis and missed diagnosis. Upon confirmed diagnosis, patients should receive aggressive chemoradiotherapy as first-line treatment. Furthermore, immunotherapy and targeted therapy should be considered as potential adjunctive therapeutic options.

## Data Availability

The datasets presented in this study can be found in online repositories. The names of the repository/repositories and accession number(s) can be found in the article/supplementary material.
